# Multi‐Bioinspired MOF Delivery Systems from Microfluidics for Tumor Multimodal Therapy

**DOI:** 10.1002/advs.202303818

**Published:** 2023-10-18

**Authors:** Qingfei Zhang, Gaizhen Kuang, Hanbing Wang, Yuanjin Zhao, Jia Wei, Luoran Shang

**Affiliations:** ^1^ Department of Rheumatology and Immunology Nanjing Drum Tower Hospital School of Biological Science and Medical Engineering Southeast University Nanjing 210096 China; ^2^ Oujiang Laboratory (Zhejiang Lab for Regenerative Medicine, Vision, and Brain Health) Wenzhou Institute University of Chinese Academy of Sciences Wenzhou 325001 China; ^3^ The Comprehensive Cancer Centre Nanjing Drum Tower Hospital The Affiliated Hospital of Medical School Nanjing University Nanjing 210008 China; ^4^ Shanghai Xuhui Central Hospital Zhongshan‐Xuhui Hospital and the Shanghai Key Laboratory of Medical Epigenetics International Co‐laboratory of Medical Epigenetics and Metabolism (Ministry of Science and Technology), Institutes of Biomedical Sciences Fudan University Shanghai 200032 China

**Keywords:** chemotherapy, ferroptosis, metal–organic framework, microfluidics, photodynamic therapy

## Abstract

Metal–organic framework (MOF)‐based drug delivery systems have demonstrated values in oncotherapy. Current research endeavors are centralized on the functionality enrichment of featured MOF materials with designed versatility for synergistic multimodal treatments. Here, inspired by the multifarious biological functions including ferroptosis pattern, porphyrins, and cancer cell membrane (CCM) camouflage technique, novel multi‐biomimetic MOF nanocarriers from microfluidics are prepared. The Fe^3+^, meso‐tetra(4‐carboxyphenyl)porphine and oxaliplatin prodrug are incorporated into one MOF nano‐system (named FeTPt), which is further cloaked by CCM to obtain a “Trojan Horse”‐like vehicle (FeTPt@CCM). Owing to the functionalization with CCM, FeTPt@CCM can target and accumulate at the tumor site via homologous binding. After being internalized by cancer cells, FeTPt@CCM can be activated by a Fenton‐like reaction as well as a redox reaction between Fe^3+^ and glutathione and hydrogen peroxide to generate hydroxyl radical and oxygen. Thus, the nano‐platform effectively initiates ferroptosis and improves photodynamic therapy performance. Along with the Pt‐drug chemotherapy, the nano‐platform exhibits synergistic multimodal actions for inhibiting cancer cell proliferation in vitro and suppressing tumor growth in vivo. These features indicate that such a versatile biomimetic MOF delivery system from microfluidics has great potential for synergistic cancer treatment.

## Graphical abstract

A “Trojan Horse”‐like system is successfully fabricated from microfluidics for synergistic tumor chemotherapy, ferroptosis, and photodynamic therapy. The designed system demonstrates satisfactory efficiency in killing tumor cells in vitro and suppressing tumor growth in vivo through the combined multimode actions. These studies reveal that the “Trojan Horse”‐like system is a promising synergistic therapy approach for cancer therapy.

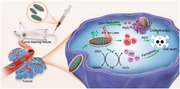

## 1 Introduction

Cancer is a leading cause of death worldwide, seriously threatening human health.^[^ ^1^ ^]^ Chemotherapy, as one of the main traditional therapy methods, has been largely indispensable in cancer treatment.^[^ ^2^ ^]^ Typically, platinum‐based chemotherapeutics (cisplatin, oxaliplatin, carboplatin, etc.) are widely employed for dealing with various cancers.^[^ ^3^ ^]^ Unfortunately, due to the inevitable adverse effects, drug resistance, and the complexity and heterogeneity of malignancies, chemotherapy alone often cannot achieve a satisfactory therapeutic outcome.^[^ ^4^ ^]^ In view of this, a variety of nanotherapeutic platforms have been designed for multimodal treatment, aimed at amplifying the therapeutic effect.^[^ ^5^ ^]^ Specifically, metal–organic frameworks (MOFs) serve as promising drug nanocarriers owing to their high porosity and large surface area, together with the synthetic tunability that enables the integration of phototherapies, chemodynamic therapy (CDT), ferroptosis therapy, and other therapy strategies.^[^ ^6^ ^]^ Despite the rapid progress in this field, a judicious design of the MOFs‐based nanocarrier satisfying multifaceted requirements including controlled drug loading and release, therapeutic efficacy, and biocompatibility remains challenging.^[^ ^7^ ^]^ Besides, high batch‐to‐batch variation of commonly developed drug‐laden MOFs renders them lacking flexibility, precision, and versatility in specific elaborative applications.^[^ ^8^ ^]^ Therefore, the development of novel multifunctional, stable, and accurate MOF delivery systems for efficient multimodal therapy is still highly sought after.

**Figure 1:**
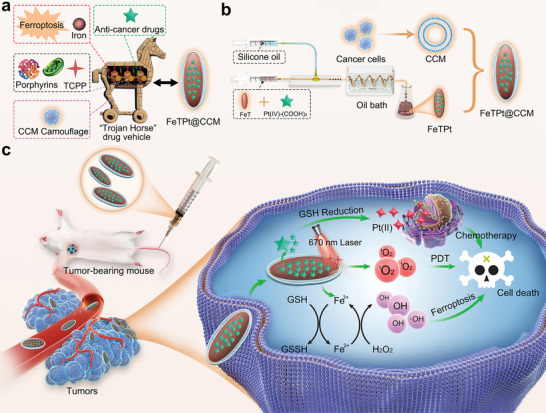
Schematic illustration of the preparation of the FeTPt@CCM and its capability of synergistic chemotherapy, PDT, and ferroptosis therapy. a) The design of the multi‐bioinspired“Trojan Horse” drug vehicle. b) The preparation processes of FeTPt through microfluidics, and the generation of FeTPt@CCM through CCM coating. c) After intravenous injection, FeTPt@CCM can efficiently accumulate at the tumor site and enter cancer cells by endocytosis. Subsequently, the Pt(IV)‐(COOH)_2_ was gradually released to the cytoplasm and reduced to high cytotoxic Pt(II) by GSH for chemotherapy. The released Fe^3+^ reacted with GSH and H_2_O_2_ through the redox reaction, which could promote GSH depletion and ^•^OH generation for initiating ferroptosis. In addition, the TCPP in the FeTPt@CCM could be activated by 670 nm light irradiation to produce ^1^O_2_ for PDT. Thus, the FeTPt@CCM exhibits synergistic therapeutic effects by combining chemotherapy, ferroptosis therapy, and PDT.

In this paper, inspired by the multifarious biological functions of cells, we proposed a multi‐biomimetic MOF delivery system with high stability and tumor‐targeting ability for combined ferroptosis therapy, chemotherapy, and photodynamic therapy (PDT), as schemed in **Figure** **1**. Ferroptosis is a recently identified kind of programmed cell death mode induced by iron‐dependent lipid peroxidation and the accumulation of reactive oxygen species (ROS).^[^ ^9^ ^]^ Ferroptosis is involved in the occurrence and development of cancer; inducing ferroptosis of cancer cells has been investigated as an effective treatment option.^[^ ^10^ ^]^ Porphyrin is a class of organic compounds with conjugated macrocycle structures.^[^ ^11^ ^]^ Benefiting from their superior photophysical and photochemical properties, porphyrins and derivatives have been utilized in phototherapies (PDT or photothermal therapy (PTT)) against cancers.^[^ ^12^ ^]^ Thus, an iron‐ and porphyrin‐integrated nanocarrier should facilitate combined therapy. Moreover, homologous cancer cell membrane (CCM) camouflage technology provides an excellent solution to improve the tumor‐targeting ability of the nanocarrier.^[^ ^13^ ^]^ Therefore, it is conceivable that by combining the above techniques in one nano‐system, a novel multimodal therapy platform with desired features can be developed. However, the construction of such a platform is still in its infancy, and its application for tumor combination therapy remains elusive.

Herein, we developed a multifunctional MOF nano‐system loaded with required therapeutic agents via droplet microfluidics and camouflaged it with CCM for synergistic tumor multimodal therapy. Microfluidic technology has emerged as a universal platform for synthesizing various nano‐ or micro‐sized materials; these materials have found widespread biomedical applications such as disease treatment, drug delivery, and tissue engineering.^[^ ^14^ ^]^ Compared with conventional bulk approaches, microfluidic‐assisted material production has significant advantages including controllable size and morphology, higher reproducibility, and improved encapsulation efficiency.^[^ ^15^ ^]^ Therefore, stable and accurate fabrication of drug‐laden MOFs could be obtained by microfluidic technology for required multimodal therapy. In this work, by leveraging a two‐step microfluidic emulsification strategy, Fe^3+^ and organic ligands (meso‐tetra(4‐carboxyphenyl)porphine (TCPP)) reacted to form the complex MOF (named FeT), which was subsequently decorated with oxaliplatin prodrug through ligand exchange, thus generating Pt‐drug‐laden MOFs (denoted as FeTPt). To improve the stability and tumor‐targeting ability, FeTPt was further coated with CCM to obtain a multi‐bioinspired “Trojan Horse”‐like vehicle (FeTPt@CCM). The FeTPt@CCM, incorporated with polynary therapeutic agents, possessed extraordinary properties including ferroptosis‐inducing ability, photodynamic performance, and chemotherapeutic activity. Consequently, as demonstrated via in vitro and in vivo investigations, the as‐prepared “Trojan Horse” vehicle exerted multimodal actions to augment cancer therapeutic efficacy. All the results implied that the microfluidics‐derived multi‐bioinspired MOF delivery systems could be applied as versatile and ideal vehicles, and hold great promise in multimodal tumor therapy.

## 2 Results and Discussion

### 2.1 Preparation and Characterization of FeTPt

In a typical experiment, FeT MOF was first synthesized from a coaxial capillary microfluidic system. The system consisted of two syringe pumps, one glass capillary microfluidic chip, one heating reaction zone, and one sample collection zone (Figure S1, Supporting Information). A pre‐mixed solution of Fe^3+^ and TCPP served as the inner phase and was pumped through the inner tube into the microfluidic chip, and the outer phase of silicone oil was pumped through the outer tube in the same direction. The solution of reactants was dispersed into droplets in the oil phase and then pumped into a micro‐pipeline. Thus, the microdroplets acted as microreactors where the reaction happened and passed through the heating bath, generating the reddish‐brown FeT MOF. After the separation of FeT, it was mixed with Pt(IV)‐(COOH)_2_ (Figures S2–S6, Supporting Information) and the reactants underwent the same reaction process through the above droplet microfluidic system to form the Pt‐drug‐laden MOF, i.e., FeTPt.

**Figure 2:**
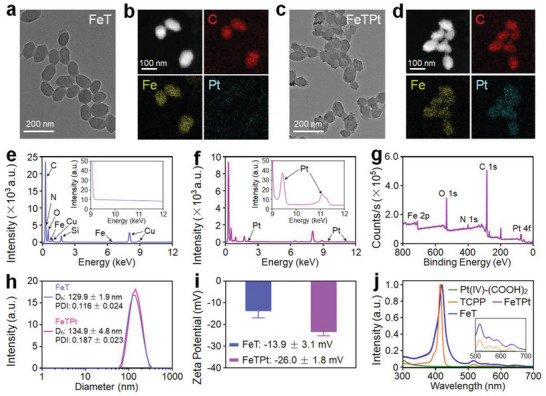
Preparation and characterization of FeTPt. a–d) TEM image and elemental mapping of (a,b) FeT and (c,d) FeTPt. e,f) EDS spectra of (e) FeT and (f) FeTPt. g) XPS spectrum of FeTPt. h) Hydrodynamic size distributions and PDI analyses of FeT and FeTPt. i) Zeta potentials of FeT and FeTPt. j) UV–vis spectra of Pt(IV)‐(COOH)_2_, TCPP, FeT, and FeTPt.

As characterized by transmission electron microscopy (TEM), both FeT and FeTPt exhibited uniform morphology (**Figure** **2**a,c). The TEM mapping assay provided a straightforward to see the integration of Pt‐prodrug (Pt(IV)‐(COOH)_2_) into the FeTPt (Figure 2b,d–f). Different from the FeT, the Pt element was uniformly distributed on the surface of the FeTPt. X‐ray photoelectron spectroscopy (XPS) revealed the binding energies of Fe *2p* and Pt *4f* were appealed in FeTPt, implying that Fe and Pt elements were doped in the MOF (Figure 2g). In comparison, the Pt element was not shown in the spectrum of FeT (Figure S7, Supporting Information). After the decoration of Pt‐prodrug, the average hydrodynamic diameter of FeTPt was slightly increased (Figure 2h). Because more carboxyl from Pt‐prodrug was introduced to the FeTPt, the zeta potential of FeTPt was obviously decreased, as compared to FeT (Figure 2i). As recorded by UV–vis spectra, the characteristic peak at 415 of TCPP had a redshift to 420 nm after the formation of FeT and FeTPt (Figure 2j). Besides, the other absorption peaks of TCPP at 516, 552, 585, and 643 nm are also shown in the spectra of FeT and FeTPt, confirming the composition of MOFs. As detected by inductively coupled plasma mass spectrometry (ICP‐MS), the Fe and Pt loading content in the FeTPt were 7.87 ± 0.50 and 4.43 ± 0.11 wt.% (Table S1, Supporting Information), respectively. Collectively, these findings demonstrated the successful preparation of FeTPt.

### 2.2 Catalase‐Like, GSH Peroxidase‐Like, and PDT/CDT‐Like Reactivities of FeTPt

**Figure 3:**
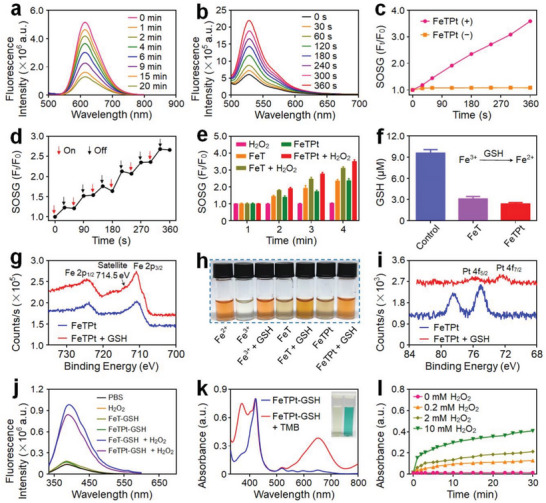
GSH peroxidase‐like, PDT/CDT‐like, and catalase‐like reactivities of FeTPt. a) Fluorescence spectra of [Ru(dpp)_3_]Cl_2_ incubated with FeTPt and H_2_O_2_ for different time intervals. b) Fluorescence spectra of SOSG incubated with FeTPt and irradiated with 670 nm laser irradiation for different times. c) The fluorescence changes of SOSG at 528 nm after incubation with FeTPt with or without irradiation. d) The fluorescence changes of SOSG on the response of On‐Off irradiation for FeTPt. e) Relative fluorescence intensity of SOSG at 528 nm incubated with FeTPt plus H_2_O_2_ and with irradiation. f) GSH concentrations after different treatments. g) XPS spectra of Fe *2p* in FeTPt before and after incubation with GSH. h) The chromogenic reaction of *o*‐phenanthroline after different treatments. i) XPS spectra of Pt *4f* in FeTPt before and after incubation with GSH. j) Fluorescence spectra of TPA after different treatments. k) UV–vis spectra and photo (inset) of the catalyzed oxidation of TMB treated with FeTPt plus H_2_O_2_. l) The absorbance changes of TMB treated with FeTPt and GSH plus different concentrations of H_2_O_2_.

It has been reported that Fe^3+^ could catalyze hydrogen peroxide (H_2_O_2_) to produce oxygen (O_2_), which could quench the fluorescence of tris(4,7‐diphenyl‐1,10‐phenanthroline)ruthenium(II) dichloride ([Ru(dpp)_3_]Cl_2_).^[^ ^16^ ^]^ As indicated in **Figure** **3**a, as the co‐incubation period of FeTPt and H_2_O_2_ increased from 0 to 20 min, the fluorescence intensity of [Ru(dpp)_3_]Cl_2_ rapidly decreased. By contrast, when [Ru(dpp)_3_]Cl_2_ was incubated with PBS and H_2_O_2_, the fluorescence spectra negligibly changed (Figure S8a, Supporting Information). Besides, the fluorescence intensity of [Ru(dpp)_3_]Cl_2_ incubated with FeTPt and without adding H_2_O_2_ also has inappreciable changes (Figure S8b, Supporting Information). These findings demonstrated FeTPt's catalase‐like ability to generate O_2_. O_2_ plays a crucial role in PDT.^[^ ^17^ ^]^ During PDT, the photosensitizer undergoes a transition from its ground state to an excited state. In the excited state, the photosensitizer can react with nearby O_2_ molecules to produce singlet oxygen (^1^O_2_) and other reactive oxygen species (ROS).^[^ ^18^ ^]^ These ROS possess strong oxidative properties, capable of attacking cell membranes, mitochondria, nuclei, and other intracellular structures, leading to cell damage and apoptosis.^[^ ^19^ ^]^ Therefore, the O_2_ generation resulting from the FeTPt‐catalyzed H_2_O_2_ reaction can enhance PDT performance. As one of the porphyrin derivatives, TCPP could act as a photosensitizer by absorbing photons and interacting with O_2_ to generate ^1^O_2_ for PDT.^[^ ^20^ ^]^ Therefore, the FeTPt containing TCPP is expected to possess the capability to generate ^1^O_2_ under light irradiation. To verify this, a ^1^O_2_ sensor green (SOSG) detector was applied to assess the ^1^O_2_ generation ability of FeTPt. As shown in Figure 3b and Figure S9a (Supporting Information), when the FeT or FeTPt was irradiated by a 670 nm laser, the intensity of the SOSG fluorescence significantly elevated with the elongation of time. By contrast, the FeTPt without irradiation did not induce variations in the fluorescence spectra of SOSG (Figure S9b, Supporting Information). Besides, the relative fluorescence intensity (*F*_t_/*F*_0_) of SOSG evidently increased with a nearly linear relationship with the exposure time, reaching ≈3.6 times after 360 s, suggesting high photostability for continuous ^1^O_2_ production (Figure 3c). Moreover, FeTPt could precisely respond to “On‐Off” irradiation and retain the capacity of repeatedly producing ^1^O_2_ with several irradiation cycles (Figure 3d). It is noteworthy that FeTPt with H_2_O_2_ under laser irradiation could produce much higher ^1^O_2_ than that without H_2_O_2_ (Figure 3e; Figure S10, Supporting Information), suggesting that the self‐supply of H_2_O_2_ could enhance ^1^O_2_ generation. H_2_O_2_ is a crucial signaling molecule in various physiological processes including cell growth, proliferation, and aging.^[^ ^21^ ^]^ In comparison to normal tissues, tumors often exhibit higher levels of H_2_O_2_.^[^ ^22^ ^]^ The generation rate of H_2_O_2_ in cancer cells could reach up to 0.5 nmol 10^−4^ cells h^−1^.^[^ ^23^ ^]^ Thus, the endogenous H_2_O_2_ within the tumor could be catalytically decomposed by catalase‐like properties of FeTPt to generate O_2_ in situ, thereby enhancing ^1^O_2_ generation for PDT.^[^ ^24^ ^]^

Furthermore, FeTPt exhibits glutathione (GSH) peroxidase‐like activity. As demonstrated in Figure 3f, after 6 h of co‐incubation with FeTPt, the content of GSH in solution rapidly declined by more than 75%, which is critical for preventing the rapid clearance of ROS by intracellular reduction components like GSH. Meanwhile, along with the oxidative depletion of GSH, the Fe^3+^ and Pt(IV) prodrugs in FeTPt were converted to Fe^2+^ and Pt(II) species, respectively. The XPS spectrum revealed a new peak at ≈714.5 eV that corresponded to Fe 2*p*, providing clear proof of Fe^2+^ production (Figure 3g). The generation of Fe^2+^ was also verified by adding *o*‐phenanthroline, which could form orange complexes with Fe^2+^. Thus, after the reduction of Fe^3+^ by GSH, the solution containing FeTPt obviously turned orange (Figure 3h), which was consistent with the UV–vis spectra showing that the broad absorbance peak appeared from 350 nm to 560 nm after the complex formation between Fe^2+^ and *o*‐phenanthroline (Figure S11, Supporting Information). Additionally, after the incubation with GSH, the characteristic peaks of Pt(IV) at 78.5 and 75.0 eV have evidently changed to 75.8 and 72.3 eV, respectively, indicating the degradation of Pt(IV) to Pt(II) (Figure 3i).

The production of Fe^2+^ could exhibit Fenton‐like activities to catalyze H_2_O_2_ decomposition to generate hydroxyl radical (^•^OH), which could be detected after the reaction with terephthalate (TPA).^[^ ^25^ ^]^ In the presence of TPA, the reaction product of FeTPt and GSH reacted with H_2_O_2_ could induce a dramatic increase in fluorescence intensity of TPA (Figure 3j), implying the generation of large quantities of ^•^OH in the solution. In addition, the time‐dependent formation of ^•^OH was also observed using the chromogenic reaction of tetramethylbenzidine (TMB), which would be oxidized to produce a unique blue color.^[^ ^26^ ^]^ As illustrated in Figure 3k and Figure S12 (Supporting Information), the characteristic absorbance peak at 652 nm caused by oxidized TMB suggested that FeTPt could trigger the decomposition of H_2_O_2_. The increased absorption of TMB at 652 nm was correlated with the increase in H_2_O_2_ concentration (Figure 3l). These findings revealed that FeTPt could execute multiple types of catalytic processes including GSH peroxidase‐like, PDT/CDT‐like, and catalase‐like reactivities to induce radical storms.

### 2.3 Preparation and Characterization of FeTPt@CCM

**Figure 4:**
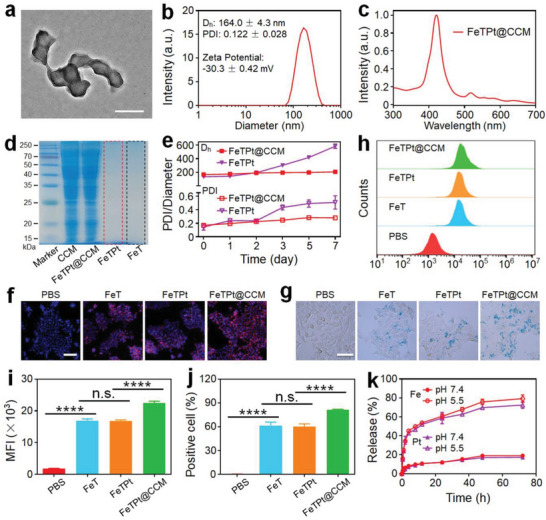
Preparation and characterization of FeTPt@CCM. a) TEM image of FeTPt@CCM. Scale bar = 200 nm. b) Hydrodynamic size distribution, PDI analysis, and Zeta potential of FeTPt@CCM. c) UV–vis spectra of FeTPt@CCM. d) SDS‐PEGA protein electrophoresis analyses of CCM, FeT, FeTPt, and FeTPt@CCM. e) Hydrodynamic size distributions and PDI analyses of FeTPt and FeTPt@CCM in 7 days. f) CLSM images and g) Prussian blue staining of 4T1 cells incubated with FeT, FeTPt, and FeTPt@CCM for 8 h. f: Scale bar = 50 µm. g: Scale bar = 25 µm. h–j) Flow cytometric analysis (h) of 4T1 cells after incubation with FeT, FeTPt, and FeTPt@CCM for 8 h and the corresponding mean fluorescence intensity (MFI) analysis (i) and cellular uptake efficiency analysis (j). k) Pt and Fe release profiles of FeTPt@CCM under different conditions.

To improve the stability and enhance the targeting ability of FeTPt, it was coated with the breast cancer cell membrane (CCM) (Figure S13, Supporting Information), and the CCM‐coated FeTPt (FeTPt@CCM) was carefully characterized. As observed in **Figure** **4**a, a layer of CCM is on the surface of FeTPt@CCM. The diameter and zeta potential of FeTPt@CCM separately increased and decreased to 164 nm and −30.3 mV, as compared to FeTPt (Figure 4b). The characteristic peaks of TCPP in FeTPt@CCM remained after the CCM coating (Figure 4c). Besides, almost identical electrophoretic phenotypes between CCM and FeTPt@CCM lysate could be seen in Figure 4d, verifying the successful camouflage of CCM and that the membrane proteins were not obviously affected. After the CCM coating, the changes in hydrodynamic size and polydispersity (PDI) of FeTPt@CCM were inconspicuous over the period of 7 days (Figure 4e; Figure S14, Supporting Information), while for FeT and FeTPt, the aggregation of nanoparticles could be detected after 2 days incubation. In addition, we measured the UV–vis spectra changes of the nanoparticles on different days. As shown in Figure S15 (Supporting Information), the UV–vis absorption peak intensities of FeT and FeTPt were obviously decreased with the extensions of incubation time because of the gradual aggregation of the nanoparticles. By contrast, the absorption peak of FeTPt@CCM was little influenced. These results collectively suggested that membrane coating could improve the stability of FeTPt@CCM, and thus might contribute to its performance against cancer located in intricate microenvironments.

After these characterizations, we assessed the cell uptake efficiency of FeTPt@CCM by breast cancer 4T1 cells compared with FeT and FeTPt via fluorescence imaging, Prussian blue staining, and flow cytometry analysis (Figure 4f–j; Figure S16, Supporting Information). As shown in Figure 4f,g and Figure S16 (Supporting Information), much more red or blue fluorescence could be observed from the cells incubated with FeTPt@CCM compared to those of FeT and FeTPt treatment groups. Meanwhile, the curve of the flow cytometry and the quantitative mean fluorescence intensity (MFI) analyses displayed consistent results, in which a remarkably higher fluorescence intensity could be observed after the cells were incubated with FeTPt@CCM (Figure 4h,i). The cellular uptake efficiencies of FeT, FeTPt, and FeTPt@CCM were 61.2%, 60.3%, and 81.3% (Figure 4j), respectively. These results verified that the cellular uptake efficiency of FeTPt@CCM was enhanced compared with that of FeT and FeTPt, which was attributed to the good homotypic targeting ability of FeTPt@CCM after the CCM camouflage. The Fe and Pt loading content in the FeTPt@CCM was detected by an ICP‐MS and calculated to be ≈5.77 ± 0.48 and 3.19 ± 0.41 wt.% (Table S1, Supporting Information), respectively. Subsequently, the release behaviors of FeTPt@CCM were studied under different conditions. It could be observed that Fe and Pt were slowly released from FeTPt@CCM at pH 7.4, and only 19.05% and 17.26% of which were released after 72 h (Figure 4k), respectively. However, under the acid environment at pH 5.5, the release contents were significantly increased to 79.29% and 72.51%. Besides, the release rate of FeTPt was slightly faster than that of FeTPt@CCM (Figure S17a, Supporting Information). This could be attributed to the CCM that retarded the drug release from the nanoparticles. In addition, the release behaviors of FeTPt and FeTPt@CCM in Dulbecco's modified eagle medium (DMEM) containing fetal bovine serum (10%) indicated that the drug release rates of FeTPt were obviously faster than FeTPt@CCM (Figure S17b, Supporting Information), implying that CCM coating would improve the stability of nanoparticles. These results indicated the good stability and tumor targeting ability as well as effective drug release behavior of FeTPt@CCM.

### 2.4 In Vitro Anti‐Cancer Ability of FeTPt@CCM

**Figure 5:**
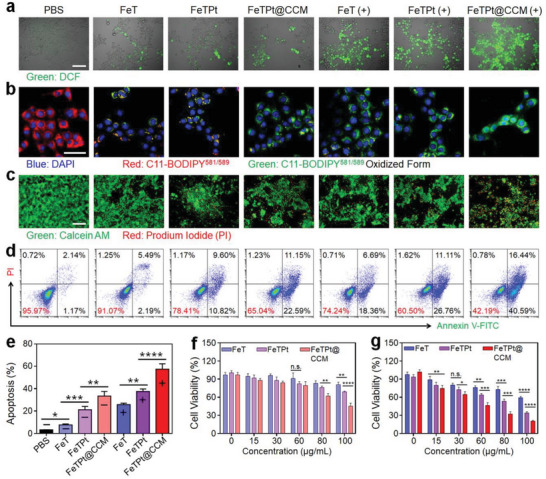
In vitro anti‐cancer ability of FeTPt@CCM. a) Fluorescence images of 4T1 cells with various treatments and incubated with DCFH‐DA. Scale bar = 50 µm. b) CLSM images of 4T1 cells with various treatments and incubated with C11‐BODIPY^581/591^. Scale bar = 50 µm. c) Live/dead staining of 4T1 cells after various treatments for 24 h and stained by Calcein AM and PI. Scale bar = 100 µm. d) Flow cytometry analysis of tumor cells after various treatments for 24 h. e) Corresponding apoptosis rates of various treatment groups. f,g) CCK8 assay of tumor cells after various treatments without (f) or with (g) irradiation. (−) indicates groups without irradiation and (+) indicates groups with irradiation. All the cell experiments had three independent replicates (*n* = 3). Data are presented as the mean ± SD. n. s.: no significance, ^*^ *p* < 0.05, ^**^ *p* < 0.01, ^***^ *p* < 0.001, and ^****^ *p* < 0.0001.

The excellent homologous cell targeting ability of FeTPt@CCM and the quickly released Fe and Pt‐drug under acid conditions could facilitate synergistic PDT, ferroptosis therapy, and chemotherapy for cancer treatment. To evaluate the PDT ability of FeTPt@CCM, 4T1 cells treated with different drugs were received with or without irradiation. Subsequently, DCFH‐DA was employed to detect ROS generation. As indicated in **Figure** **5**a and Figure S18 (Supporting Information), the green fluorescence from 2′,7′‐dichlorodihydrofluorescein (DCF) in the groups without irradiation could be ascribed to the Fenton‐like reaction. In addition, bright green fluorescence in the cells treated with various drugs with irradiation could be seen because of more ROS generation. Notably, the FeTPt@CCM + irradiation group displayed the strongest green fluorescence, verifying the photo‐induced ROS generation and the potential PDT ability of FeTPt@CCM. On the other hand, C11‐BODIPY^581/591^ was used as an indicator to investigate the intracellular levels of lipid peroxide (LPO, a ferroptosis marker) induced by FeTPt@CCM. As observed from confocal laser scanning microscopy (CLSM) images (Figure 5b; Figure S19, Supporting Information), 4T1 cells incubated with different drugs displayed different degrees of LPO. Without laser irradiation, cells in the FeTPt@CCM group exhibited stronger green fluorescence from the oxidized C11‐BODIPY^581/591^ due to the more cell uptake of FeTPt@CCM and more ^•^OH generation compared to FeT and FeTPt groups. The fluorescence intensities were further enhanced in the cells treated with different drugs with laser irradiation because of the ^1^O_2_ production, and the FeTPt@CCM group displayed the strongest green fluorescence, suggesting the potent ferroptosis‐inducing ability of FeTPt@CCM.

Based on the above mechanisms, the therapeutic efficiency of FeTPt@CCM was tested against 4T1 cells in vitro. Live/dead cell analysis based on Calcein AM (green)/propidium iodide (PI, red) co‐staining was applied to directly evaluate the cancer cell‐killing effect of FeTPt@CCM. As observed in Figure 5c and Figure S20 (Supporting Information), FeTPt displayed better anti‐cancer efficacy than FeT with or without irradiation because of the additional Pt‐drug chemotherapy. Notably, the FeTPt@CCM exhibited appreciably greater therapeutic efficiency than FeT and FeTPt did. With irradiation, the largest quantity of dead cells could be seen in the FeTPt@CCM treatment group. The apoptosis analysis results were consistent with the live/dead study. The apoptosis rates of cells in the FeT, FeTPt, and FeTPt@CCM groups without irradiation were 7.63 ± 0.85%, 21.19 ± 2.99%, and 33.40 ± 4.15%, respectively (Figure 5d,e). As expected, the different formula had a better cell‐killing effect against cancer cells under laser irradiation, and the FeTPt@CCM + irradiation group had the best efficacy with an apoptosis rate of 57.4 ± 4.81%. Furthermore, the CCK8 detection also revealed much higher cell toxicity of FeTPt@CCM against cancer cells under laser irradiation (Figure 5f,g). All these results demonstrated that the FeTPt@CCM could display efficiently tumor cell‐killing efficacy via synergetic chemotherapy, ferroptosis, and PDT.

### 2.5 In Vivo Imaging and Biodistribution

**Figure 6:**
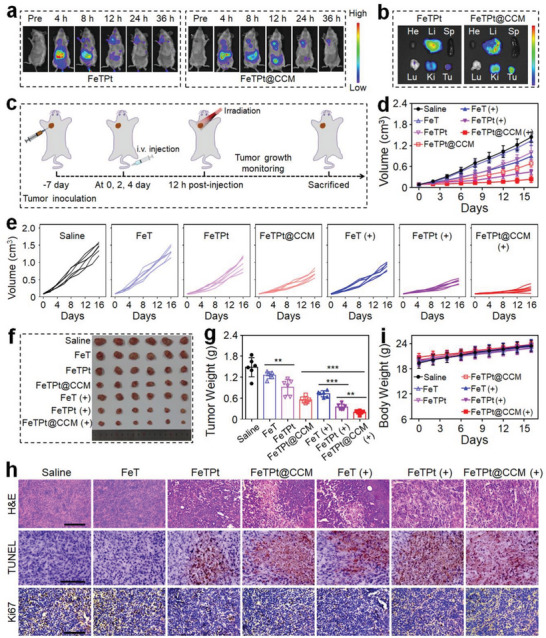
In vivo anti‐cancer ability of FeTPt@CCM. a) In vivo fluorescence images of tumor‐bearing mice (n = 3) after intravenous injection of FeTPt and FeTPt@CCM. b) Fluorescence images of major organs and tumors at 12 h after intravenous injection of FeTPt and FeTPt@CCM. c) Schematic illustration of the therapeutic schedule. d,e) Tumor growth curves after various treatments. f) Image of tumors isolated from tumor‐bearing mice at the end of the treatment. g) Weight of tumors collected from mice at the end of the treatment. h) H&E, TUNEL, and Ki67 staining of tumor tissues after different treatments. Scale bar = 100 µm. i) Body weights of mice during the treatments. (−) indicates groups without irradiation and (+) indicates groups with irradiation. Each experiment group has six mice (*n* = 6). Data are presented as the mean ± SD. ^**^ *p* < 0.01 and ^***^ *p* < 0.001.

The excellent in vitro anti‐cancer effect of FeTPt@CCM laid the foundation for the further investigation of its in vivo tumor therapeutic performance. First, the in vivo bio‐distribution of nanoagents was measured by an in vivo imaging system (IVIS) after intravenous injection (*i.v*.) of FeTPt or FeTPt@CCM labeled with indocyanine green (ICG) into mice bearing breast tumors. The fluorescence signal at the tumor site gradually increased for both FeTPt and FeTPt@CCM‐treated mice and reached their maxima after 12 h injection, indicating efficient tumor accumulation of the nanoagents (**Figure** **6**a; Figure S21, Supporting Information). The FeTPt@CCM was mainly distributed at the tumor, kidney, and liver (Figure 6b), and the FeTPt@CCM treatment showed much more tumor accumulation than the FeTPt group. This should be attributed to the CCM camouflage that endowed FeTPt@CCM with homotypic targeting ability. The fluorescence intensity of FeTPt@CCM then gradually decreased over the course of the following 12 h. In contrast, significantly lower signals were displayed at tumor sites in the FeTPt‐treated group, which may be related to the fast clearance of the immune system and lack of tumor‐targeting ability. In addition, the bio‐distributions of FeTPt and FeTPt@CCM were also evaluated in tumor‐bearing mice. Quantification of the distribution profiles showed that the Pt contents in tumors had higher values at 12 h compared with other time points (Figure S22, Supporting Information). Of note, the FeTPt@CCM accumulated significantly more in the tumors than FeTPt, with the intratumoral Pt content 1.53‐fold higher 12 h post‐injection. The biodistribution results also indicated effective tumor‐targeting ability and accumulation of FeTPt@CCM.

### 2.6 In Vivo Anti‐Cancer Ability of FeTPt@CCM

Subsequently, the in vivo multimode tumor inhibition ability of FeTPt@CCM was evaluated after intravenous injection and with laser irradiation. The treatment schedule of FeTPt@CCM was schematized in Figure 6c. As per the previous reports, the 671 nm red light is located in the “therapeutic window” (650–900 nm), and the tissue penetration deep of the 671 nm red light could reach up to 16 mm.^[^ ^27^ ^]^ Thus, the orthotopic breast tumor model was initially established as a relatively superficial tumor, allowing penetration by the applied 670 nm red light for in vivo antitumor efficacy evaluation. Without irradiation, the FeTPt@CCM displayed better therapeutic efficacy compared to FeT and FeTPt, which should be attributed to the more effective tumor accumulation of FeTPt@CCM and the actions of chemotherapy and ferroptosis (Figure 6d–g). As expected, the tumor‐suppressive performance of FeTPt@CCM was further enhanced when the mice were exposed to laser irradiation because of the additional PDT effect. At the end of monitoring, the FeTPt@CCM group had the smallest tumor volume and the lightest tumor weight (Figure 6f,g). It is worth noting that the treatment of free oxaliplatin could retard the growth of tumors in the first 6 days, but the tumors were out of control during the following observation period (Figure S23a–c, Supporting Information). These data confirmed the superior therapeutic efficiency of FeTPt@CCM based on the synergistic actions of chemotherapy, ferroptosis, and PDT.

In addition, the multimode therapeutic efficacy was revealed by hematoxylin and eosin (H&E), terminal‐deoxynucleoitidyl transferase‐mediated nick end labeling (TUNEL), and Ki67 staining. It could be seen that FeTPt@CCM treatment with irradiation induced the most severe histological destruction, the highest degree of apoptosis, and the least proliferative activity of tumor tissues (Figure 6h). Along with the treatment, the body weights in each MOF system treatment group were increased, while noticeable body weight loss in the oxaliplatin‐treated group could be observed due to its systemic toxicity (Figure 6i; Figure S23d, Supporting Information). At the end of the experiment, the hematological analysis demonstrated that the WBC, RBC, PLT, and HGB had negligible differences in all MOF treatment groups (Figure S24a–d, Supporting Information). The blood biochemical measurement also revealed that the ALT and AST did not show noticeable changes in all MOF treatments (Figure S24e,f, Supporting Information). Besides, no apparent pathologic abnormalities were seen in the main organs stained with H&E in all MOF treatment groups (Figure S25, Supporting Information). These results validated the good biosafety of the developed MOF delivery systems.

## 3 Conclusion

In summary, we developed a multi‐bioinspired MOF delivery system (FeTPt@CCM) from droplet microfluidic technology for synergistic cancer chemotherapy, ferroptosis therapy, and PDT. The biomimetic “Trojan Horse”‐like vehicle (FeTPt@CCM) with CCM camouflage exhibited good biological stability and tumor homologous targeting ability. After accumulation in the tumor site, the biomimetic FeTPt@CCM first entered cells through endocytosis, then decomposed in the cytoplasm to release oxaliplatin prodrug and Fe^3+^. The oxaliplatin prodrug could be reduced to oxaliplatin by intracellular GSH for inducing chemotherapy. Besides, the Fe^3+^ in FeTPt@CCM reacted with GSH and H_2_O_2_ through the redox reaction, which could promote GSH depletion and ^•^OH generation for initiating ferroptosis. In addition, the TCPP in the FeTPt@CCM could be activated by red light irradiation to produce ^1^O_2_, and such photodynamic performance could be enhanced via O_2_ generation from the catalase‐like activity of Fe^3+^. Thus, the combination of multimode actions of chemotherapy, ferroptosis, and PDT exhibited satisfactory efficiency in killing tumor cells in vitro and suppressing tumor growth in vivo, together with good safety. Collectively, this work provided a microfluidic strategy to prepare MOF delivery system integrated with multiple therapy modalities, broadening the research and potential application of bioinspired nanomedicine in drug delivery and oncotherapy.

Recently, cancer immunotherapy, which harnesses the body's intrinsic immune system to eliminate cancer cells, has emerged as a revolutionary and promising approach to cancer treatment.^[^ ^28^ ^]^ Owing to its easy accessibility and high abundance of various tumor‐specific antigens for antigen presentation, CCM has often been designed for the CCM‐coated nanovaccines intended for cancer immunotherapy.^[^ ^29^ ^]^ Therefore, in addition to the targeting effect of the CCM in the developed FeTPt@CCM, the possible immunostimulatory effect of the CCM may also play an important role in the superior anticancer efficacy of the FeTPt@CCM. Besides, many treatment methods including PTT, PDT, ferroptosis, chemotherapy, radiotherapy, etc. could trigger immunogenic cell death (ICD) for stimulating antitumor immunity.^[^ ^30^ ^]^ It is conceivable that the prepared FeTPt@CCM has the potential to serve as an ICD initiator for immunotherapy through a synergistic combination of chemotherapy, ferroptosis, and PDT actions. Thus, such versatile MOF offers ample research opportunities for tumor therapy and provides novel prospects for combined multimode tumor treatment.

## 4 Experimental Section

### Materials

Oxaliplatin, meso‐tetra(4‐carboxyphenyl)porphine (TCPP), benzoic acid (BA), 1,10‐phenanthroline, tetramethyl benzidine (TMB), p‐phthalic acid (PTA), anhydrous FeCl_3_, and FeCl_2_ were bought from Biochemical Technology Co., Ltd. (Shanghai, China). H_2_O_2_ solution (30%) and succinic anhydride were bought from Aladdin. [Ru(dpp)_3_]Cl_2_ was purchased from Bide Pharmatech Co., Ltd. (Shanghai, China). H&E staining kits were purchased from Solarbio Science & Technology Co., Ltd. (Beijing, China). Singlet oxygen (^1^O_2_) sensor green (SOSG) was purchased from Meilun Biotechnology Co., Ltd (Dalian, China). DCFH‐DA, glutathione (GSH) detection kit, annexin V‐FITC/PI apoptosis detection kit, and CCK8 assay kit were purchased from Beyotime Biotechnology Co., Ltd. (Shanghai, China).

### Synthesis of Dicarboxylic Oxaliplatin Prodrug (Pt(IV)‐(COOH)_2_)

First, oxaliplatin was oxidized by H_2_O_2_ (30%) to obtain the dihydroxyl oxaliplatin prodrug (Pt(IV)). Briefly, 20 mL aqueous solution containing 1.0 g of oxaliplatin was added with H_2_O_2_ (10 mL), followed by stirring at 50 °C for 1 day to get the Pt(IV). ^1^H nuclear magnetic resonance spectroscopy (^1^H NMR) (DMSO‐d_6_, ppm) of oxaliplatin (Figures S2 and S3, Supporting Information): NH_2_, a, a′ (6.17, 5.38); CH_2_, b, b′ (1.27, 1.03); CH_2_, c, c′ (1.83, 1.46); CH, d, d′ (2.03). ^1^H NMR (DMSO‐d_6_, ppm) of Pt(IV) (Figures S2 and S3, Supporting Information): NH_2_, a, a′ (7.70, 6.90); CH_2_, b, b′ (1.23, 1.08); CH_2_, c, c′ (1.50); CH, d, d′ (2.00).

Then, Pt(IV)‐(COOH)_2_ was synthesized via the ring‐opening reaction between Pt(IV) and succinic anhydride. In detail, succinic anhydride (50 mg, 0.5 mmol) and Pt(IV) (53.1 mg, 0.1 mmol) were added to the dried dimethyl sulfoxide (DMSO, 5 mL) before being stirred at 60 °C for 1 day. Subsequently, the reaction solution was poured into excess diethyl ether, and the obtained precipitates were washed stepwise with minimum cold acetone, ethanol, and diethyl ether to get the Pt(IV)‐(COOH)_2_. ^1^H NMR (DMSO‐d_6_, ppm) of Pt(IV)‐(COOH)_2_ (Figure S4, Supporting Information): NH_2_, a, a′ (8.14, 8.35); CH_2_, b, b′ (1.15); CH_2_, c, c′ (1.49, 1.38); CH, d, d′ (2.10, 2.12); CH_2_, e (2.58); CH_2_, f (2.38); COOH, g (12.11). ^13^C NMR (DMSO‐d_6_, ppm) of Pt(IV)‐(COOH)_2_ (Figure S5, Supporting Information): CO, a (180.12); CO, b (174.18); CO, c (163.86); CH, d (61.30); CH_2_, e (31.39); CH_2_, f (30.98); CH_2_, g (30.01); CH_2_, h (23.95). ESI‐MS (m/z) of Pt(IV)‐(COOH)_2_ (Figure S6, Supporting Information): [M─H]^−^, 630.2.

### Synthesis of FeTPt

A dimethylformamide (DMF) solution (10 mL) containing TCPP (10 mg, 0.013 mm), FeCl_3_ (18 mg, 0.11 mm), and BA (280 mg, 2.29 mm) was injected into a droplet microfluidics flow‐reaction device through a syringe pump. At a constant flow rate ratio (Q_silicone oil_:Q_reactant_ = 16:1), the solution of reactants was dispersed into uniform micro‐droplets. These micro‐droplets passed along the reaction silicone coil (2 × 3 mm) which was immersed in an oil bath (*T* = 90 °C). The prune solution could be collected in a vial after 5 h residence. The sample was rinsed using n‐hexane and ethanol three times before being centrifuged for 30 min at 12 000 rpm. The as‐prepared MOFs (FeT) were dissolved in DMF (5 mL). Subsequently, FeTPt was synthesized via a ligand exchange strategy between Pt(IV)‐(COOH)_2_ and FeT. Briefly, a DMF solution containing FeT was mixed with Pt(IV)‐(COOH)_2_ (9.5 mg, 0.016 mmol), and the solution was injected in a droplet microfluidics flow‐reaction device through a syringe pump. Under the flow rate ratio of 16:1 (Q_silicone oil_:Q_reactant_), the micro‐droplets were reacted at 70 °C for 24 h. Then, the prepared FeTPt was washed with ethanol and n‐hexane three times before being centrifuged at 12 000 rpm for 0.5 h. Finally, for further usage, the FeTPt was stored in PBS at 4 °C.

### Preparation of Cancer Cell Membranes (CCM)

The preparation of CCM has been slightly modified based on previous reports. Briefly, breast cancer 4T1 cells were digested and collected. Then, the cells were dispersed in a membrane separation buffer for 15 min at 4 °C. After that, the lysate immediately underwent repeated six‐time freezing‐thawing cycles. Next, the mixture was centrifuged for 20 min at 700 g to precipitate the pellets, and the cell membranes were produced by centrifuging the resulting supernatant for 30 min at 14 000 g. For future use, the obtained CCM was lyophilized.

### Preparation of FeTPt@CCM

The isolated CCM together with FeTPt were resuspended in PBS and stirred for 30 min. The mixture was then extruded using a Mini‐Extruder (Alabama, USA) 15 times to get FeTPt@CCM. Before being stored in PBS at −80 °C for later usage, the FeTPt@CCM was washed with PBS and harvested by centrifuging (13 000 rpm) for 20 min.

### Characterizations

A TEM (FEI Talos, USA) was applied to measure the morphology, structure, and surface elements of the prepared MOFs. UV–vis–NIR spectrometer (Agilent, CARY5000, USA) was applied to investigate the UV absorption spectra of FeT, FeTPt, and FeTPt@CCM. The zeta potentials and hydrodynamic diameters of the MOFs were recorded by DLS. The chemical elements and valence states in MOFs were analyzed by XPS.

### Protein SDS‐PAGE Electrophoresis

The samples of CCM, FTPt, and FeTPt@CCM were heated to 95–100 °C for 10 min in the loading buffer. Then, 12% sodium dodecyl SDS‐PAGE was employed to separate different samples. Subsequently, the gel was stained with Coomassie blue for 0.5 h. After that, the gel was washed with a solution containing methanol (25%, v/v), glacial acetic acid (8%, v/v), and ddH_2_O (67%, v/v) 3 times to remove the blue background. Finally, the gel was imaged by a digital camera.

### Drug Release In Vitro

Briefly, FeTPt or FeTPt@CCM (0.2 mg mL^−1^) in PBS was introduced to a dialysis bag (MwCO = 1000) at pH 7.4 or 5.5. The dialysis unit was then placed in 19 mL of the release media and subjected to constant shaking (100 rpm) at 37 s°C. After timed incubation, 1 mL of the released dialysis solution was removed following timed incubation, and an equal volume of the fresh medium was added to the dialysis system to keep the volume constant. The released Fe and Pt were measured by ICP‐MS. By using the same method, the release behaviors of FeTPt or FeTPt@CCM in DMEM at pH 7.4 were measured.

### Oxygen (O_2_) Detection

[Ru(dpp)_3_]Cl_2_, whose fluorescence could be dynamically quenched by O_2_, was used as an indicator to detect the O_2_ generation. The FeTPt (50 µg mL^−1^) solution containing [Ru(dpp)_3_]Cl_2_ (2 µm) was added with 10 mm H_2_O_2_. Then, under 470 nm excitation, the fluorescence spectrogram of [Ru(dpp)_3_]Cl_2_ (500–800 nm) was measured. The FeTPt (50 µg mL^−1^) solution containing [Ru(dpp)_3_]Cl_2_ (2 µm) without adding H_2_O_2_ was set as a control. Besides, the [Ru(dpp)_3_]Cl_2_ (2 µm) solution containing H_2_O_2_ and without adding FeTPt was also set as a control.

### ^1^O_2_ Generation

A SOSG agent was employed to detect ^1^O_2_ generation. Briefly, SOSG (5 µm) was added to a solution of FeTPt (50 µg mL^−1^) or FeT (50 µg mL^−1^). After the solution was irradiated (670 nm, 100 mW cm^−2^) for indicated time intervals, the fluorescence spectra of SOSG from 500 to 700 nm were measured (Ex: 480 nm, Em: 525 nm) by a fluorescence spectrophotometer. The ^1^O_2_ generation efficiency was further examined by calculating *F*_t_/*F*_0_ at 525 nm, where *F*_0_ stands for the starting fluorescence intensity and *F*_t_ is the fluorescence intensity following exposure to radiation at various times.

To explore the ^1^O_2_ generation affected by O_2_ self‐supplementation based on MOF's catalase‐like reactivity. FeTPt (50 µg mL^−1^) or FeT (50 µg mL^−1^) solution containing 5 µm SOSG and 10 mm H_2_O_2_ for 5 min was irradiated (670 nm, 100 mW cm^−2^) for different time intervals. The ability of MOF to generate ^1^O_2_ was finally assessed using the above methods.

### GSH Depletion

Briefly, FeTPt (50 µg mL^−1^) or FeT (50 µg mL^−1^) was incubated with GSH solution (10 µm) for 6 h, followed by centrifugation (13 000 rpm) for 0.5 h. Subsequently, the supernate was obtained and the concentration of GSH was detected by a GSH assay kit. The valence states of Fe and Pt elements before and after reduction were detected by XPS.

### Fe^2+^ Detection

O‐phenanthroline was used as an indicator, which could react with Fe^2+^ to form orange complexes. In comparison, the complexes between o‐phenanthroline and Fe^3+^ are colorless. Hence, the transformation of Fe^3+^ into Fe^2+^ from FeTPt or FeT could be detected by o‐phenanthroline. Briefly, 2 mL of FeTPt (50 µg mL^−1^) or FeT (50 µg mL^−1^) solution containing GSH (100 mm) was incubated in an oscillator for 6 h, before adding with o‐phenanthroline solution (100 µL, 1 mg mL^−1^). The color changes after different treatments were observed and the photograph was taken with a camera. The absorbance peak changes were detected by UV–vis–NIR spectrometer.

### ^•^OH Generation

PTA and TMB were used to detect the ^•^OH generation. Two microliters of FeTPt (50 µg mL^−1^) or FeT (50 µg mL^−1^) solution containing GSH (100 mm) was incubated in an oscillation for 6 h before being centrifugated (13 000 rpm) for 0.5 h. Then, the resulting precipitate was mixed with a 5 mm TPA solution containing NaOH (5 mm) and H_2_O_2_ (10 mm). Following 30 min of incubation, the 300 nm excitation wavelength allowed for the detection of the fluorescence spectra from 335 to 600 nm. For TMB measurement, the aforementioned precipitate was added to a solution of acetic acid solution (1 mL, 0.1 m, pH 5.0), TMB (1 mm), and various amounts of H_2_O_2_. Using a microplate reader and UV–vis spectrometer, the absorbance at 652 nm was determined following co‐incubation for various time intervals.

### Cellular Internalization

Mouse breast cancer 4T1 cells (2.5 × 10^5^) were plated in each well of a 6‐well plate. After cultivating overnight for cell attachment, FeTPt (100 µg mL^−1^), FeT (100 µg mL^−1^), or FeTPt@CCM (100 µg mL^−1^) were added to the medium and incubated for 8 h. After that, the cells were washed three times with PBS to remove the un‐endocytosed nanoparticles. They were then fixed with 4% paraformaldehyde, stained with DAPI, and finally analyzed using a CLSM system (Ex: 561 nm, Em: 659 nm). To quantify the cellular uptake efficiency of different nanoparticles, the cells incubated with different drugs were washed three times with PBS and then collected, followed by analysis via flow cytometry.

For Prussian blue staining, 4T1 cells were seeded into a 6‐well plate (2.5 × 10^5^ cells per well) and cultivated for 12 h. After that, the cells were incubated with FeTPt, FeT, or FeTPt@CCM for 8 h. Subsequently, the cells were washed three times with PBS to remove the un‐endocytosed nanoparticles. Following this, a Prussian blue staining solution was applied, and the cells were stained for 30 min. Then, after washing with PBS three times to remove the free blue dye, the blue‐stained cells were screened, fixed in 4% paraformaldehyde, and imaged under a fluorescence microscope (OLYMPUS BX53).

### Intracellular ROS Detection

A 24‐well plate containing 5 × 10^4^ 4T1 cells in each well was planted, followed by cultivating overnight. Next, the cells were added with FeT, FeTPt, or FeTPt@CCM (100 g mL^−1^) and co‐incubated for 8 h. After three times PBS washes, a fresh solution containing DCFH‐DA (10 µm) was added, followed by incubating for another 30 min. Subsequently, the cells were irradiated (670 nm, 100 mW cm^−2^) for 5 min before capturing a picture using a fluorescent microscope.

### Intracellular Lipid Peroxide

A 24‐well plate seeded with 4T1 cells (5 × 10^4^ cells per well) was co‐cultured with various treatments for 8 h. After that, the cells were irradiated under (670 nm, 100 mW cm^−2^) for 5 min and then stained with C11‐BODIPY^581/591^ (10 µm) for 0.5 h. Finally, the cells were measured under a CLSM system after being washed with PBS.

### In Vitro Anti‐Tumor Effects

For the CCK8 assay, 4000 cells per well of 96‐well plates were treated with different drug formulations with various concentrations (0, 15, 30, 60, 80, and 100 µg mL^−1^). After incubation for 8 h, the cells were irradiated (670 nm, 100 mW cm^−2^) for 5 min. After incubation for 24 h, CCK8 reagent was added to the cells and co‐incubated for a further 2 h. Finally, a microplate reader was used to measure the absorbance at 450 nm.

For live/dead staining, the cells co‐cultured with various treatments and irradiated by a 670 nm laser were co‐stained with Calcein‐AM and PI before being observed using a fluorescent microscope.

For apoptosis analysis, the cells received the same treatments as above. After that, Annexin V‐FITC and PI were used to co‐stain the cells before analyzing them via flow cytometry.

### In Vivo Imaging and Biodistribution

The animal experiments were approved by the Animal Ethics Committee of the Wenzhou Institute, University of Chinese Academy of Sciences (approval WIUCAS21031002) and complied with the recommendations of the academy's animal research guidelines. Female BALB/c mice were obtained from the Wenzhou Institute's Animal Experimental Center and used in accordance with a local ethical council (approval WIUCAS21031002). To establish the orthotopic breast tumor model, the right breast of each mouse was injected with 1 × 10^6^ of 4T1 cells to develop tumors with a volume of ≈100 mm^3^.

To investigate the in vivo imaging of FeTPt and FeTPt@CCM, the nanoparticles were first labeled with ICG. Briefly, the FeTPt (1 mg mL^−1^) was mixed with ICG (1 mg mL^−1^) in a water solution and stirred for 4 h away from light. The solution was then centrifuged at 12 000 rpm for 0.5 h to obtain the ICG labeled FeTPt, which was further coated with CCM to obtain the ICG labeled FeTPt@CCM. Then, the mice (*n* = 3) were then intravenously injected with 100 µL, 5 mg mL^−1^ of either FeTPt or FeTPt@CCM labeled with ICG. And an in vivo imaging instrument was used for observing the mice at various intervals (Ex: 780 nm, Em: 831 nm). At 12 h after injection, the major organs and tumors were collected from the mice and the fluorescence images were investigated. To evaluate the biodistribution of the nanoparticles, the tumor‐bearing mice (*n* = 3) were intravenously injected with FeTPt (3 mg Pt per kg body) or FeTPt@CCM (3 mg Pt per kg body). At specified intervals, both the tumor and major tissues were harvested and weighed. Subsequently, the samples underwent heat‐assisted treatment with concentrated nitric acid to yield a clear solution, which was then subjected to Pt content quantification using ICP‐MS.

### In Vivo Anti‐Tumor Evaluation

Female BALB/c mice bearing 4T1 tumors were separated into seven groups, each containing six mice. The intravenous injections of Saline, Oxaliplatin, FeT, FeTPt, or FeTPt@CCM were given to the animals at days 0, 2, and 4. After 12 h injection each time, the mice received laser irradiation (670 nm, 100 mW cm^−2^) for 10 min or remained untreated. The body weights and tumor sizes of mice were measured every other day. The tumor volumes were determined using the formula V = L × W^2^/2, where *L* and *W*, respectively, stand for the tumors' length and width.

### Histological Analysis and TUNEL Assay

The tumor tissues and primary organs were peeled off from the sacrificed mice after 16 days of treatment, and they were then immersed in 4% paraformaldehyde. The tissues were then cut into samples that were 5 m thick and stained with H&E and colorimetric TUNEL assay kit.

### Hematological and Blood Biochemical Analyses

The mice's blood was collected as well at the end of the experiment for hematological and blood biochemical examinations.

### Statistical Analysis

All statistical data were expressed as the mean ± standard deviations. Statistical evaluation was analyzed using unpaired Student's *t*‐test or one‐way ANOVA, and a *p*‐value < 0.05 was considered statistically significant. n. s.: no significance, ^*^ *p* < 0.05, ^**^ *p* < 0.01, ^****^ *p* < 0.0001, and ^***^ *p* < 0.001.

## Conflict of Interest

The authors declare no conflict of interest.

## Author Contributions

Y.J.Z., L.R.S., and J.W. conceived the conceptualization and designed the experiment. Q.F.Z. carried out the experiments and analyzed the data. Q.F.Z., L.R.S, and Y.J.Z. wrote the paper. G.Z.K. and H.B.W. contributed to the scientific discussion of the article.

## Supporting information

Supporting Information

## Data Availability

The data that support the findings of this study are available from the corresponding author upon reasonable request.
