# Supplementary material for: Multi‐Bioinspired MOF Delivery Systems from Microfluidics for Tumor Multimodal Therapy
Source: Adv Sci (Weinh). 2023 Oct 18;10(33):2303818. doi: 10.1002/advs.202303818 (PMC10667824; doi:10.1002/advs.202303818)
Supplement: Supplementary file 1 — Supporting Information [file ADVS-10-2303818-s001.pdf]

ADVANCED SCIENCE
Open Access

Supporting Information

for *Adv. Sci.*, DOI 10.1002/advs.202303818

Multi-Bioinspired MOF Delivery Systems from Microfluidics for Tumor Multimodal Therapy

Qingfei Zhang, Gaizhen Kuang, Hanbing Wang, Yuanjin Zhao, Jia Wei* and Luoran Shang**

Supporting Information

Multi-Bioinspired MOF Delivery Systems from Microfluidics for Tumor Multimodal Therapy

Qingfei Zhang, Gaizhen Kuang, Hanbing Wang, Yuanjin Zhao, Jia Wei*, Luoran Shang**

Dr. Q. Zhang, Dr. G. Kuang, Prof. L. Shang, Prof. Y. Zhao
Department of Rheumatology and Immunology, Nanjing Drum Tower Hospital, School of Biological Science and Medical Engineering, Southeast University, Nanjing 210096, China
E-mail: yjzhao@seu.edu.cn

Dr. Q. Zhang, Dr. G. Kuang, Prof. Y. Zhao
Oujiang Laboratory (Zhejiang Lab for Regenerative Medicine, Vision and Brain Health), Wenzhou Institute, University of Chinese Academy of Sciences, Wenzhou 325001, China

Dr. H. Wang, Prof. J. Wei
The Comprehensive Cancer Centre, Nanjing Drum Tower Hospital, The Affiliated Hospital of Medical School, Nanjing University, Nanjing 210096, China
E-mail: jiawei99@nju.edu.cn

Prof. L. Shang
Shanghai Xuhui Central Hospital, Zhongshan-Xuhui Hospital, and the Shanghai Key Laboratory of Medical Epigenetics, International Co-laboratory of Medical Epigenetics and Metabolism (Ministry of Science and Technology, Institutes of Biomedical Sciences), Fudan University, Shanghai 200032, China
E-mail: luoranshang@fudan.edu.cn

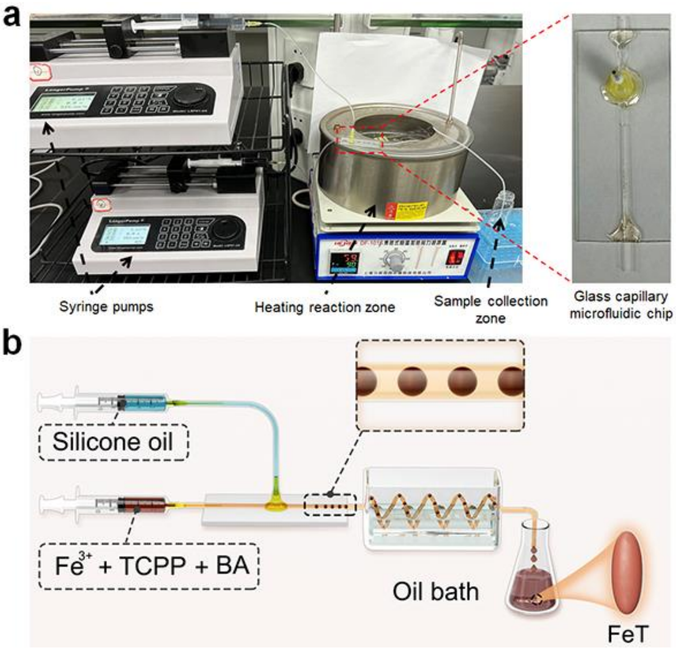

Figure S1. (a) Photographs of the microfluidic platform for the preparation of MOFs; the right image shows the capillary microfluidic chip. (b) Schematic illustration of the microfluidic platform for the preparation of FeT.

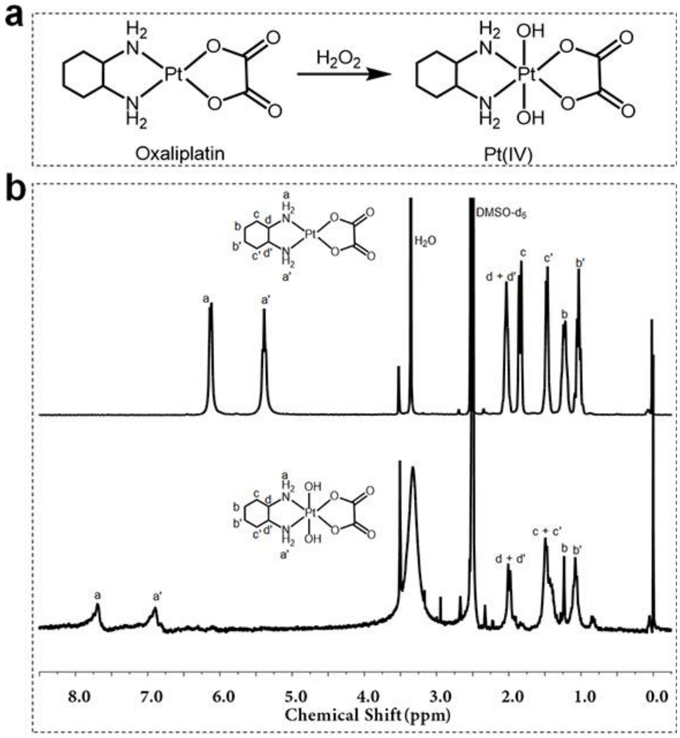

Figure S2. (a) The synthetic process of Pt(IV). (b) ^1H NMR spectra of Oxaliplatin and Pt(IV) in DMSO-d_6.

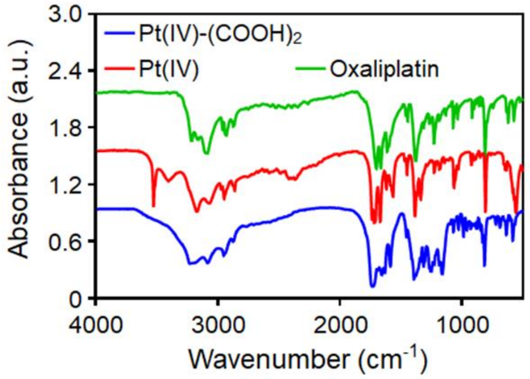

Figure S3. The FT-IR spectra of Oxaliplatin, Pt(IV) and Pt(IV)-$(COOH)_2$.

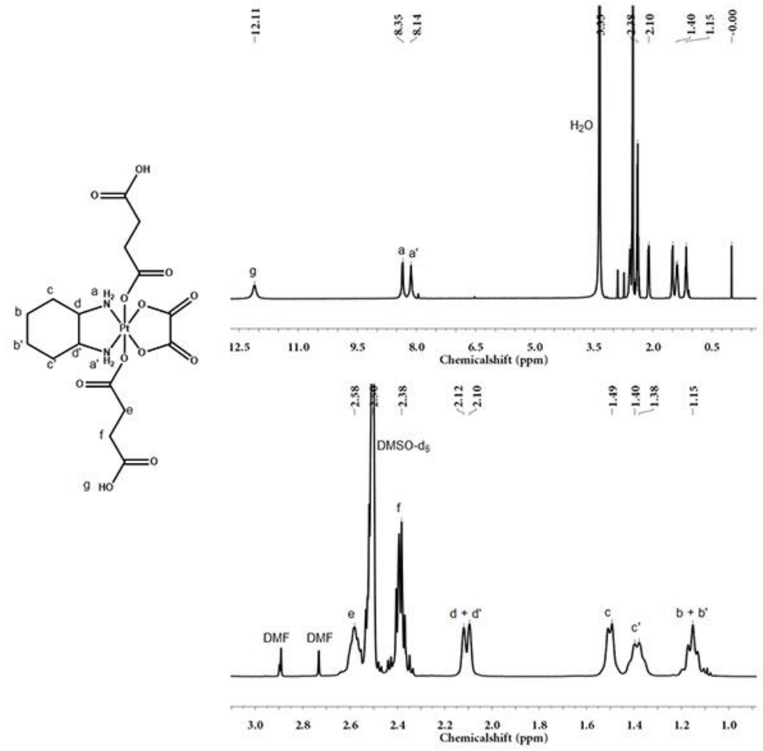

Figure S4. The ^{1}H NMR spectra of Pt(IV)-$(COOH)_2$ in DMSO-d_6.

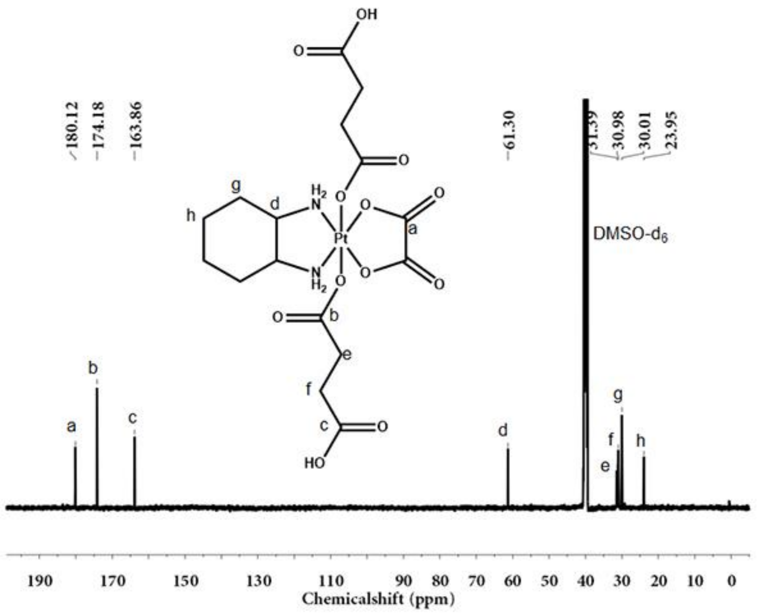

Figure S5. The ^{13}C NMR spectrum of Pt(IV)-$(COOH)_2$ in DMSO-d_6.

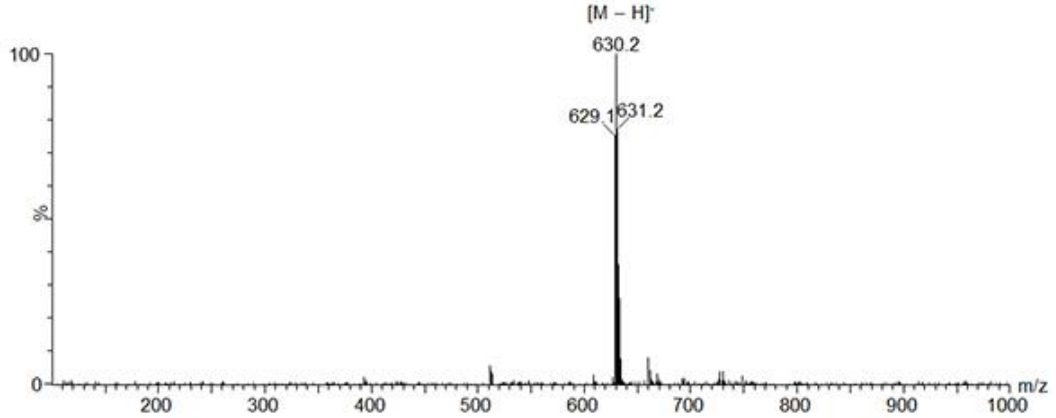

Figure S6. ESI-MS spectrum of Pt(IV)-$(COOH)_2$.

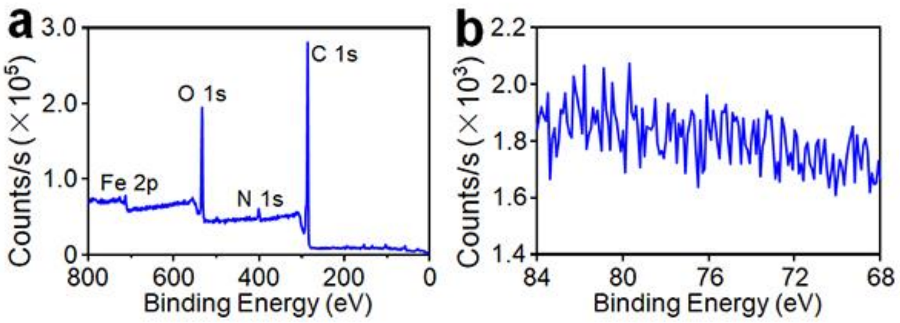

Figure S7. XPS spectra of (a) FeT and (b) Pt *4f*.

Table S1. The Fe and Pt contents in different nanoparticles.

	FeTPt	FeTPt@CCM
Fe	7.87 ± 0.50 wt%	5.77 ± 0.48 wt%
Pt	4.43 ± 0.11 wt%	3.19 ± 0.41 wt%

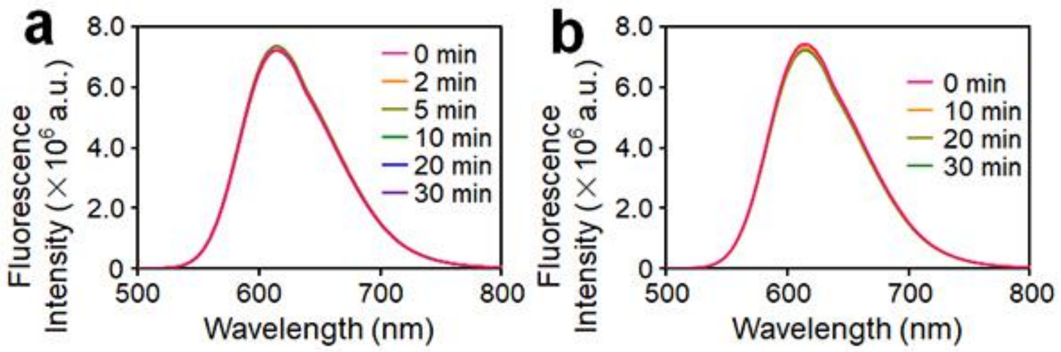

Figure S8. (a) Fluorescence spectra of $[Ru(dpp)_3]Cl_2$ incubated with PBS and H_2O_2 for different time intervals. (b) Fluorescence spectra of $[Ru(dpp)_3]Cl_2$ incubated with FeTPt and without adding H_2O_2 for different time intervals.

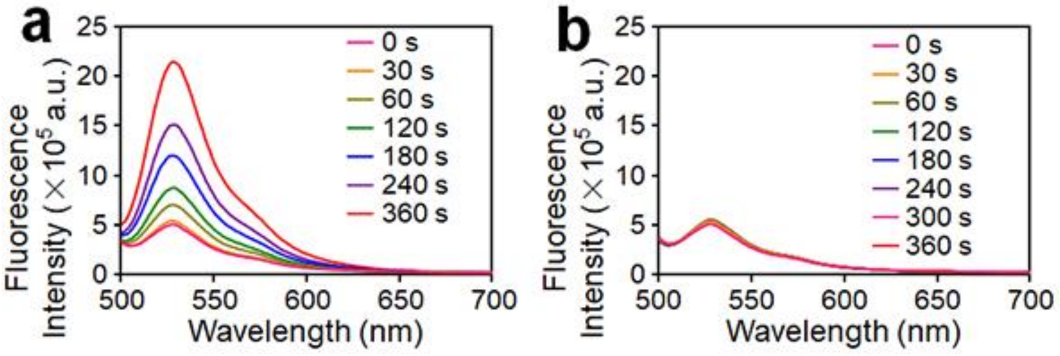

Figure S9. (a) Fluorescence spectra of SOSG incubated with FeT and irradiated with a 670 nm laser for different times. (b) Fluorescence spectra of SOSG incubated with FeTPt and without 670 nm laser irradiation.

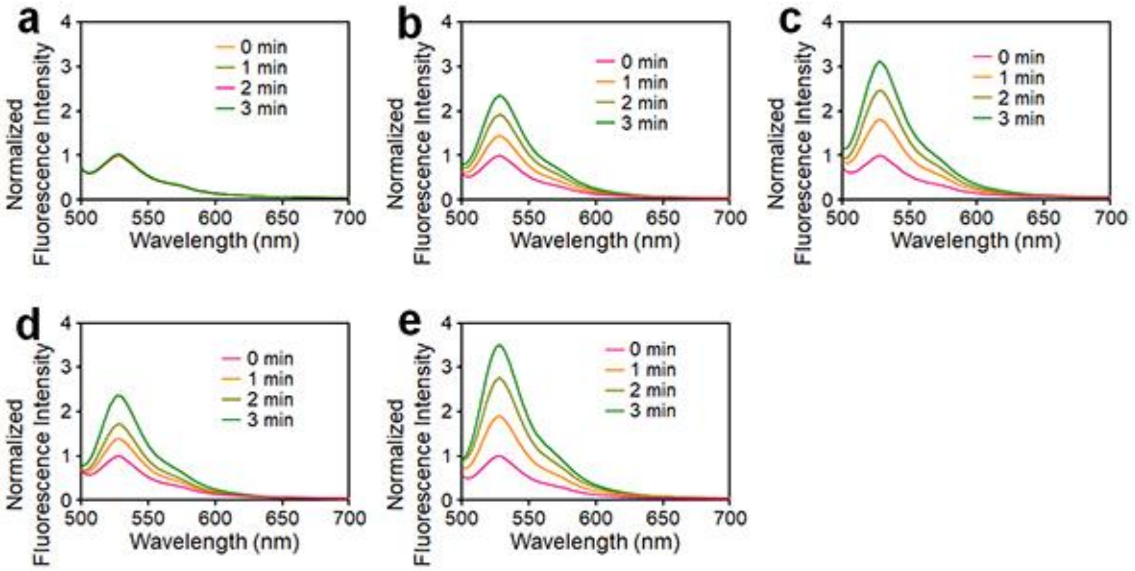

Figure S10. (a) Fluorescence spectra of SOSG incubated with H_2O_2 and with 670 nm laser irradiation. (b) Fluorescence spectra of SOSG incubated with FeT and then being irradiated by a 670 nm laser. (c) Fluorescence spectra of SOSG incubated with FeT plus H_2O_2 and then being irradiated by a 670 nm laser. (d) Fluorescence spectra of SOSG incubated with FeTPt and then being irradiated by 670 nm laser. (e) Fluorescence spectra of SOSG incubated with FeTPt plus H_2O_2 and then being irradiated by a 670 nm laser.

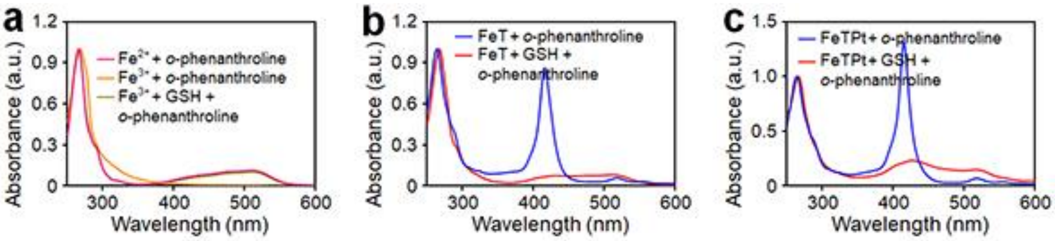

Figure S11. UV–Vis spectra of *o*-phenanthroline after different treatments.

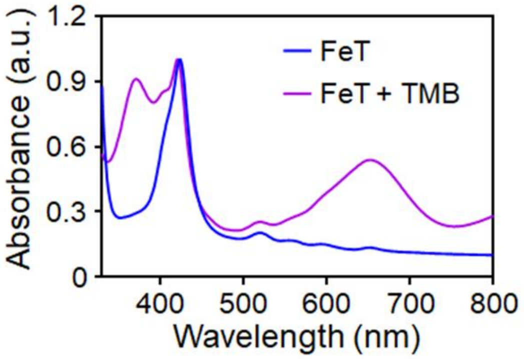

Figure S12. UV–Vis spectra of the catalyzed oxidation of TMB treated with FeT plus H_2O_2.

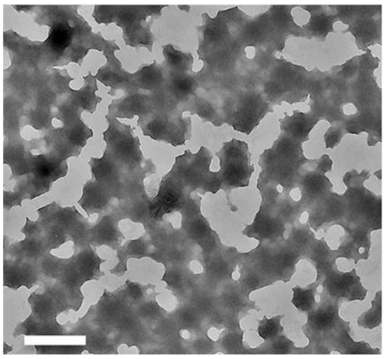

Figure S13. TEM image of CCM. Scale bar = 500 nm.

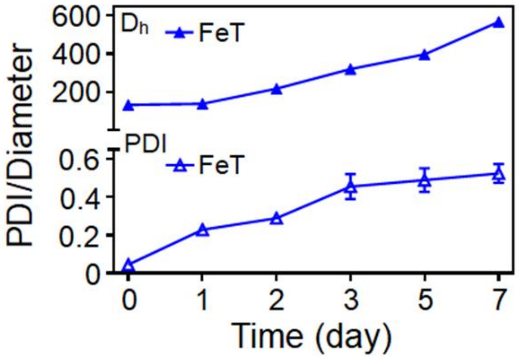

Figure S14. Hydrodynamic size distributions and PDI analyses of FeT in 7 days.

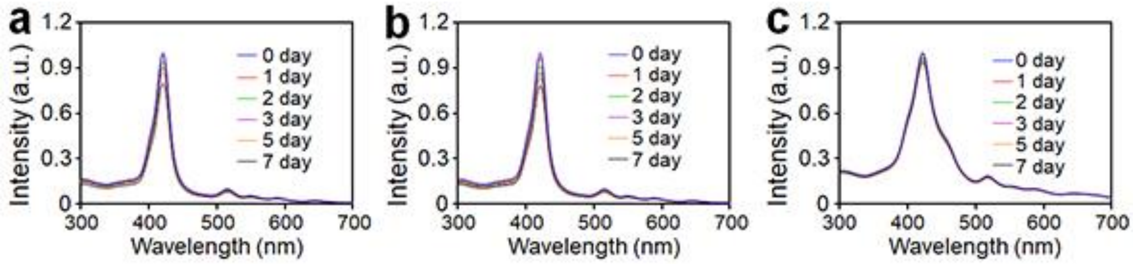

Figure S15. UV–Vis spectra changes of FeT, FeTPt, and FeTPt@CCM in 7 days.

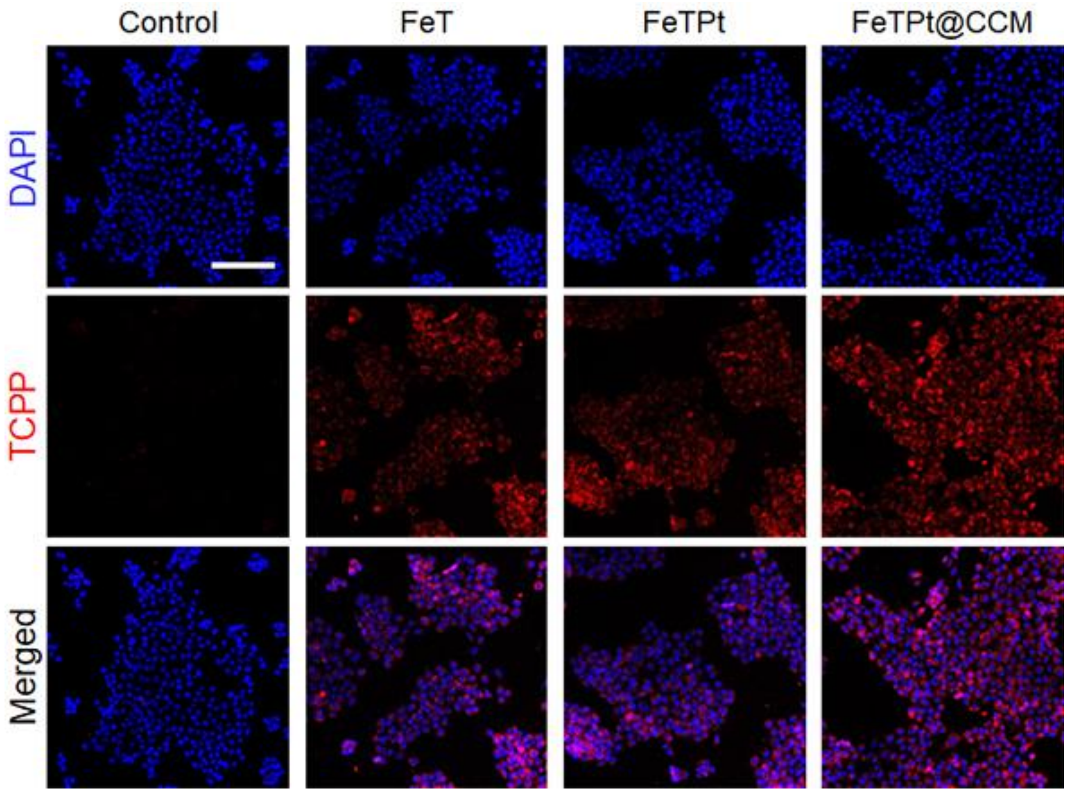

Figure S16. CLSM images of 4T1 cells incubated with FeT, FeTPt, and FeTPt@CCM for 8 h. Scale bar = 50 µm.

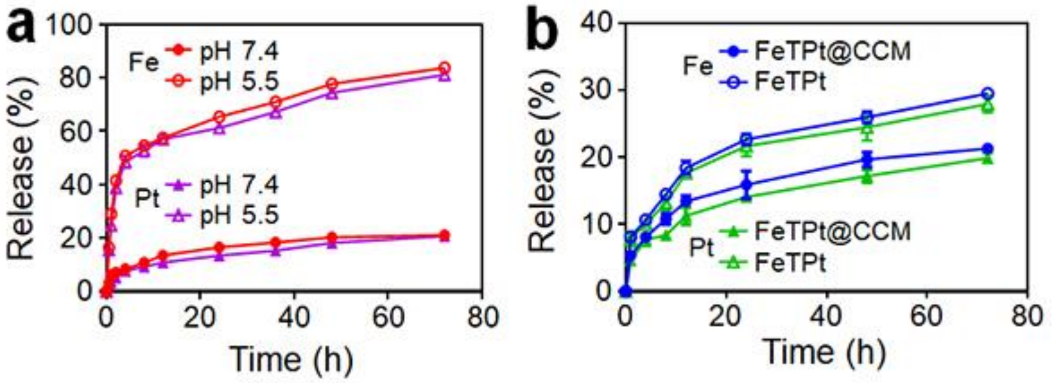

Figure S17. (a) Pt and Fe release profiles of FeTPt in PBS with different pH values. (b) Pt and Fe release profiles of FeTPt and FeTPt@CCM in DMEM containing FBS (10%).

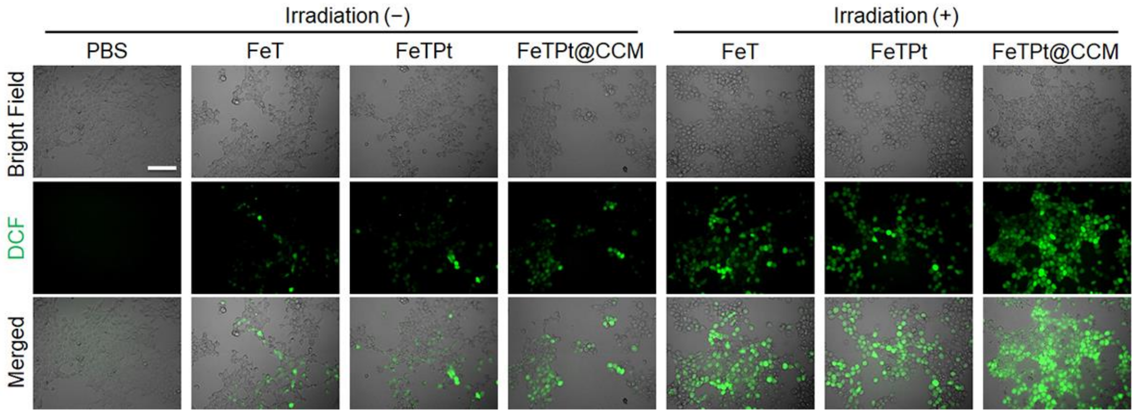

Figure S18. Fluorescence images of 4T1 cells after various treatments and incubated with DCFH-DA to detect intracellular ROS. Scale bar = 50 µm.

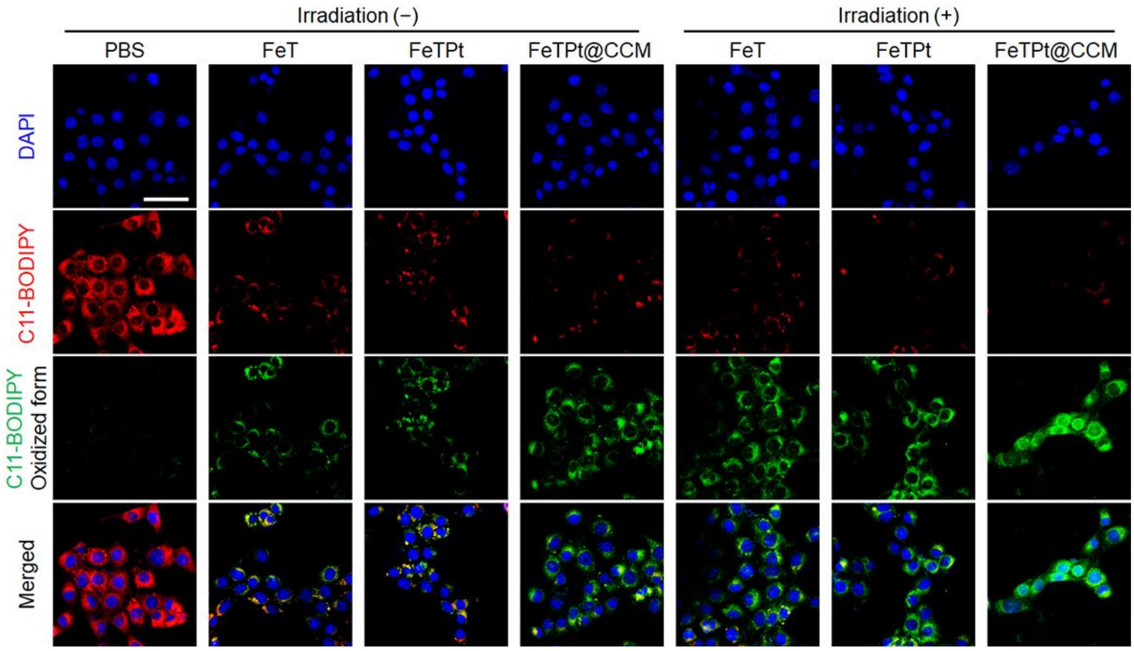

Figure S19. CLSM images of 4T1 cells after various treatments and incubated with C11-BODIPY$^{581/591}$ as a probe to detect LPO. Scale bar = 50 μm.

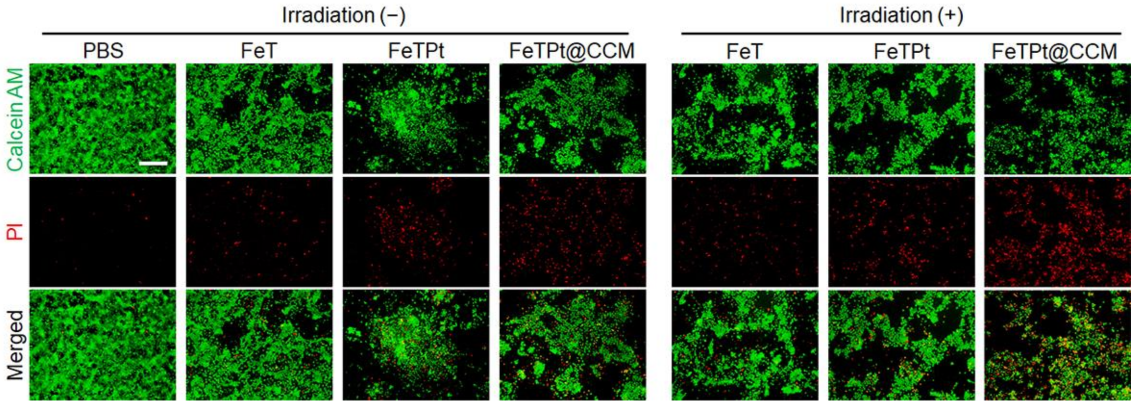

Figure S20. Live/dead staining of 4T1 cells after various treatments for 24 h and stained by Calcein AM and PI. Scale bar = 100 μm.

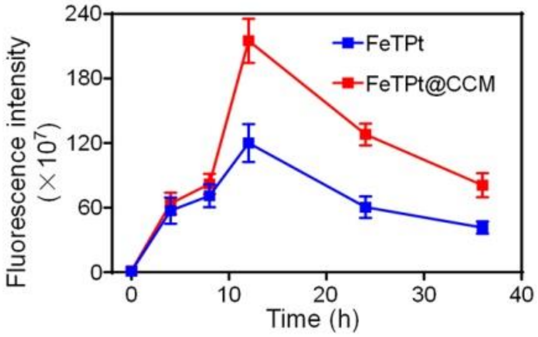

Figure S21. Quantification analyses of fluorescence intensity of tumors after injection with FeTPt or FeTPt@CCM at different time points.

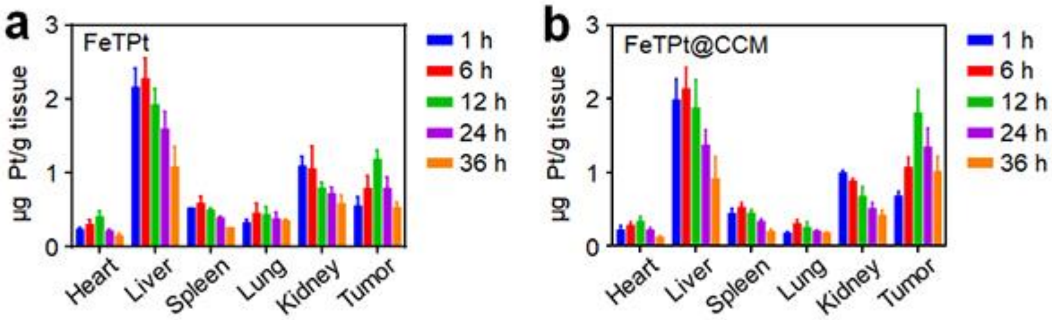

Figure S22. Biodistribution of Pt in tumors and major organs of 4T1-tumor-bearing mice after injection with FeTPt (a) or FeTPt@CCM (b) at different time points.

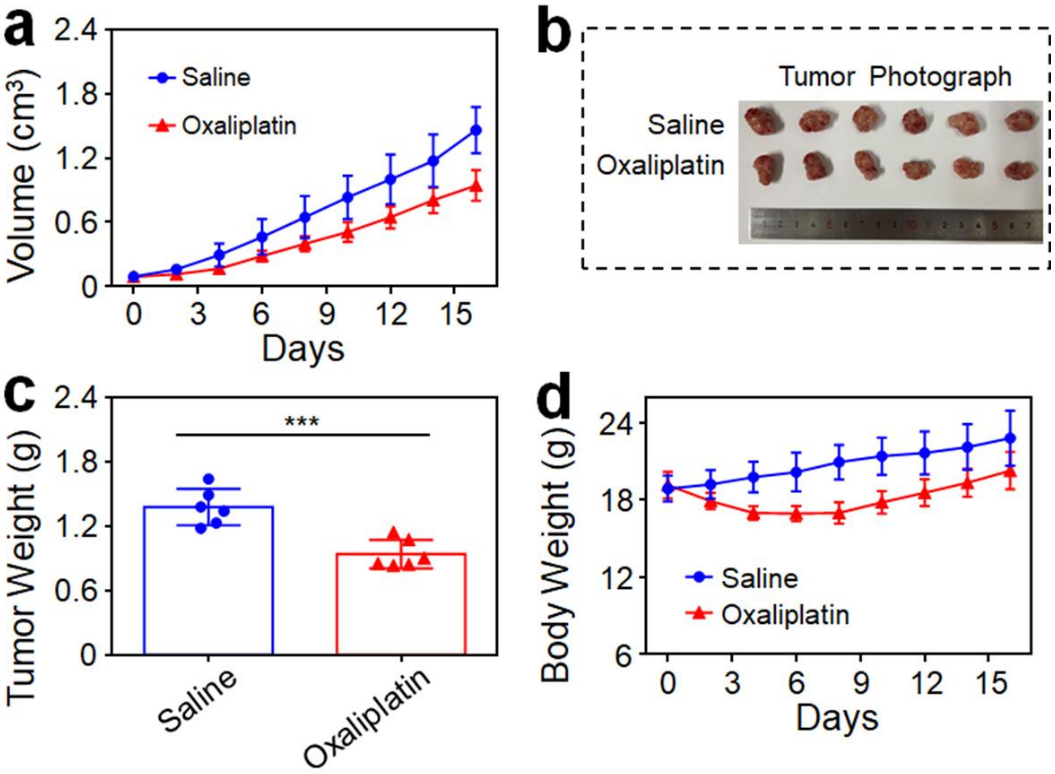

Figure S23. (a) Tumor growth curves after various treatments. (b) Image of tumors isolated from tumor-bearing mice at the end of the treatment. (c) Weight of tumors collected from mice at the end of the treatment. (d) Body weights of mice during the treatments.

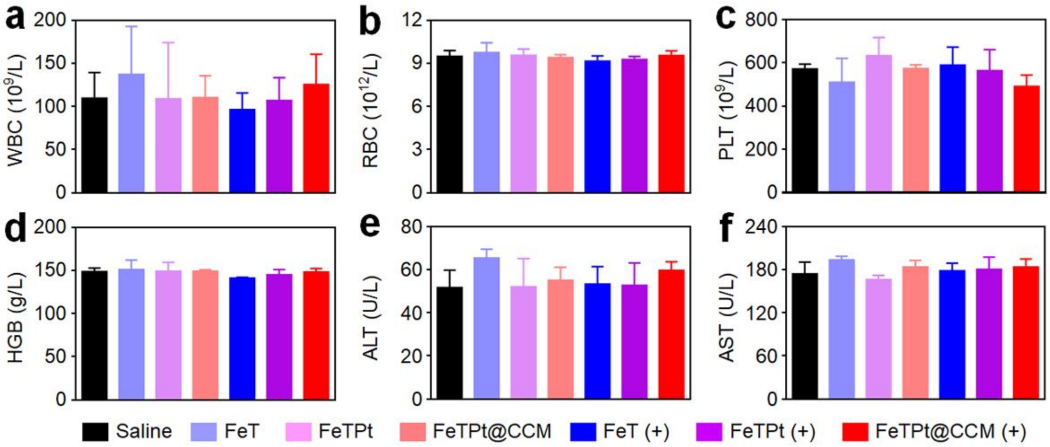

Figure S24. (a-d) Complete blood count of mice after different treatments, including (a) WBC, (b) RBC, (c) PLT, and (d) HGB. (e, f) Serum biochemistry assay of mice after different treatments, including (e) ALT and (f) AST.

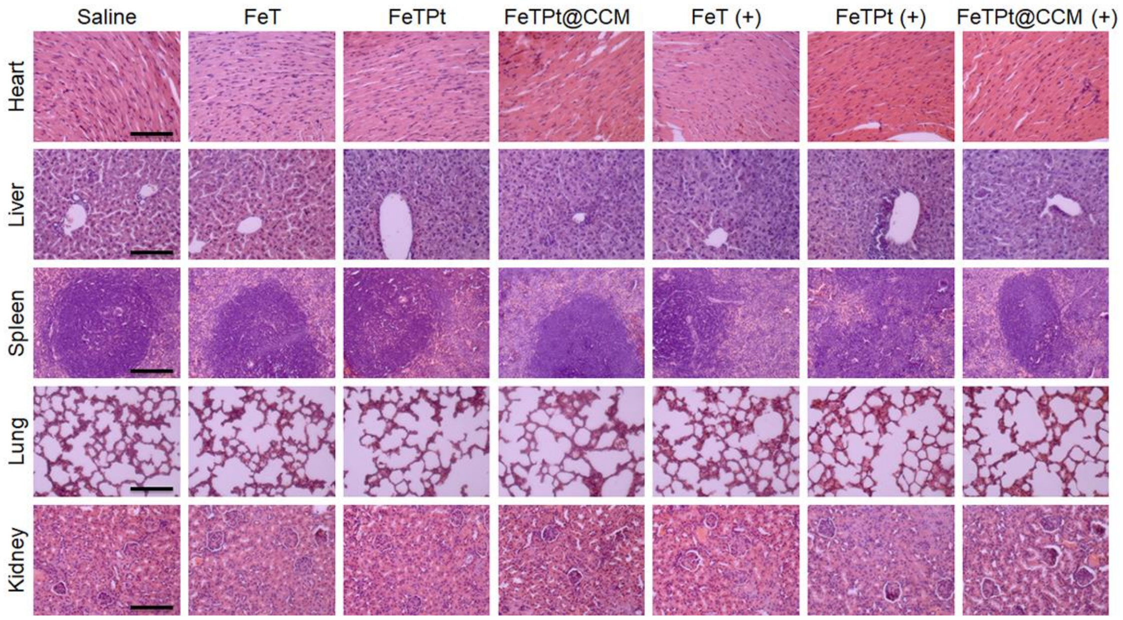

Figure S25. H&E staining of major organs collected from mice after different treatments. Scale bar = 100 µm.
